# PGxMine: Text mining for curation of PharmGKB

**Published:** 2020

**Authors:** Jake Lever, Julia M. Barbarino, Li Gong, Rachel Huddart, Katrin Sangkuhl, Ryan Whaley, Michelle Whirl-Carrillo, Mark Woon, Teri E. Klein, Russ B. Altman

**Affiliations:** 1 Department of Bioengineering, Stanford University, Stanford, CA, 94305; 2 Department of Biomedical Data Science, Stanford University, Stanford, CA, 94305; 3 Department of Medicine, Stanford University, Stanford, CA, 94305

**Keywords:** Pharmacogenomics, Biocuration, Text mining

## Abstract

Precision medicine tailors treatment to individuals personal data including differences in their genome. The Pharmacogenomics Knowledgebase (PharmGKB) provides highly curated information on the effect of genetic variation on drug response and side effects for a wide range of drugs. PharmGKB’s scientific curators triage, review and annotate a large number of papers each year but the task is challenging. We present the PGxMine resource, a text-mined resource of pharmacogenomic associations from all accessible published literature to assist in the curation of PharmGKB. We developed a supervised machine learning pipeline to extract associations between a variant (DNA and protein changes, star alleles and dbSNP identifiers) and a chemical. PGxMine covers 452 chemicals and 2,426 variants and contains 19,930 mentions of pharmacogenomic associations across 7,170 papers. An evaluation by PharmGKB curators found that 57 of the top 100 associations not found in PharmGKB led to 83 curatable papers and a further 24 associations would likely lead to curatable papers through citations. The results can be viewed at https://pgxmine.pharmgkb.org/ and code can be downloaded at https://github.com/jakelever/pgxmine.

## 1. Introduction

Precision medicine has been described as getting patients “the right drug at the right dose at the right time”.^1^ It has been shown that genetics can play a significant part in whether the drug and dose are right for that particular patient and numerous diagnostic tests have been developed to test for genetic variants related to drug efficacy.^2^ These pharmacogenomic variants encompass germline variants or somatic variants in cancer. They may affect the direct target of a drug, potentially by disrupting binding pockets, (e.g. T790M for many EGFR inhibitors^3^), the metabolizing enzymes that may affect drug concentrations, by affecting enzyme efficiency (e.g. the cytochrome P450 family of enzymes^4^), or drug transporters that may affect the disposition of the drugs and influence efficacy and toxicity.

Cataloging this expanding knowledge of pharmacogenomic variants is the goal of the PharmGKB.^5^ Launched in 2000, it collects, curates and disseminates knowledge about gene-drug associations, many of which are clinically actionable, and provides resources for both researchers and clinicians. A group of expert curators searches the scientific literature for relevant pharmacogenomic papers and add their findings to the knowledge base. However, managing the curation of a biomedical knowledge base is a very time-consuming and challenging task. As precision medicine efforts increase and the cost of sequencing decreases, more genetic biomarkers will be found that might prove to be clinically actionable. It is already a huge challenge to identify relevant papers and these technological developments will inevitably lead to an increase in the number of published papers and an increased burden on the PharmGKB curators.

Machine learning methods are used to assist in biomedical knowledge base curation in two different ways. A document-level approach attempts to identify documents that would be appropriate for curation into the knowledge base using word frequencies, metadata, and other factors. This approach was taken for the ORegAnno database,^6^ Gene Expression Database^7^ and mouse genomics.^8^ A mention-level approach attempts to extract the main associations that would be curated in the knowledge base and aggregating them to identify missing knowledge linked to curatable papers. The CIViCmine approach extracted mentions of clinically relevant cancer variants to identify the most frequently discussed that should be curated into the CIViC database.^9^ This method is linked with automated knowledge base construction methods which have been used in part to construct the STRING database of protein interactions^10^ and the CancerMine database of cancer genes^11^ directly from published literature.

To extract pharmacogenomic variants associated with specific chemicals, we propose to use a biomedical relation extraction method. The field of biomedical relation extraction focuses on extracting mentions of particular relations between entities (e.g. genes, drugs, variants, etc) in unstructured text. Dependency-parse based methods have seen great success in this area while deep learning methods achieve similar performance but face issues with overfitting.^12^ Relation extraction relies on first identifying mentions of specific entities, e.g. chemicals and variants, using entity extraction tools such as BANNER^13^ and tmVar.^14^ The PubTator resource has provided PubMed abstracts annotated with chemicals, diseases, genes, species, and variants.^15^ Numerous projects have built on top of the annotations provided by PubTator.^16,17^ Recently, the Pubtator Central resource expanded this to open-access Pubmed Central full-text articles.^18^

In this paper, we present PGxMine, a text-mined resource of pharmacogenomic associations between chemicals and variants. We build upon the PubTator Central resource and use a text-alignment method to extract specific mentions of chemicals, genes, and variants in the text. We further identify star alleles that are particularly important and frequently appear in pharmacogenomic literature (e.g. CYP2D6*2) in text. We parse and extract sentences that mention a chemical and variant and filter to enrich for sentences that likely discuss pharmacogenomic associations. We build classifiers to extract pharmacogenomic associations and apply these to all sentences accessible from PubMed abstracts and PubMed Central full-text papers. These results are then presented to the PharmGKB scientific curators to assist in their curation efforts.

## 2. Methods

**Fig. 1.**
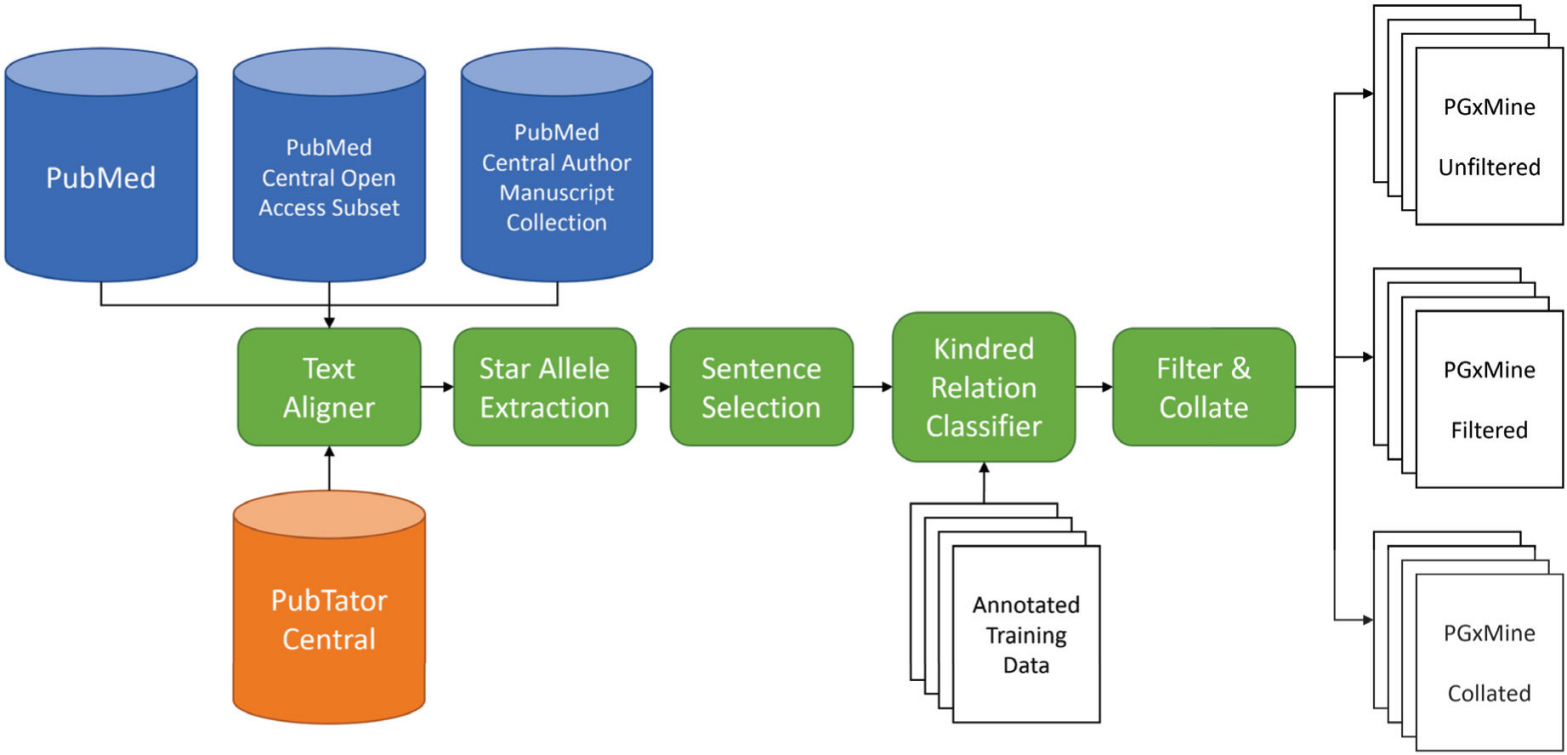
An overview of the full PGxMine system. The input data sources on the left (PubMed, etc) and PubTator Central are combined through a text alignment process to identify mentions of specific biological entities in published literature. Star Alleles (e.g. CYP2D6*2) are then found using gene annotations. Sentences are filtered using keywords to enrich for pharmacogenomic topics. A Kindred supervised classifier is then trained and applied to identify specific variant/chemical associations. These are then filtered for high probability matches and collated to produce the three output files on the right.

Figure 1 shows an overview of the approach that takes in PubTator Central entities and abstracts/articles and outputs collated sets of pharmacogenomic associations between variants and chemicals relevant to the PharmGKB project.

### 2.1. Resources

We downloaded the 21st August 2019 release of the Pubtator Central data which uses TaggerOne^19^ for chemicals, tmVar 2.0^14^ for variants and GNormPlus^20^ for genes. It provides mappings for chemical mentions to MeSH terms, genes to Entrez Gene IDs and where possible, variants to dbSNP IDs^21^ (also known as rs IDs). We used the PubRunner infrastructure to download abstracts from PubMed and full-text papers from the PubMed Open Access (PM-COA) subset and PubMed Central Author Manuscript Collection (PMCAMC) and convert to BioC format.^22^ To map chemicals in PubTator Central to the identifiers used in PharmGKB, we used DrugBank.^23^ We filtered the drug list by several categories in DrugBank to remove biological molecules that were not relevant drugs (details in supplementary materials). We also removed short terms (shorter than 4 characters). Variants were filtered using a custom set of exclusion words to remove common terms that appear to be variants but are not, e.g. for common cell-line names.

### 2.2. Text alignment

Unlike PubTator, PubTator Central does not provide the specific offsets of each biomedical entity within the text of the abstract or full paper. It provides the substring of the mention along with the PubMed ID. We used this information to align the substrings to the corresponding text in the abstract or full-text paper wherever appropriate (with example shown in Figure S1). The alignment algorithm takes each text mention and creates a regular expression to search the corresponding article text. Each regular expression starts and ends with word-boundaries so that matches wouldn’t happen inside words. We replaced each section of whitespace with a “\s+” regular expression to map to any length of whitespace. PubTator Central appears to translate the papers from Unicode to ASCII and provides ASCII mentions. Several important entities contain Unicode characters, e.g. TGF-<x so we replaced ASCII Greek letters (e.g. alpha) with a regex to map to either the ASCII text or the Greek character. These regular expressions then find candidate mappings for each text. We process the mappings largest to smallest so that no entity is mapped inside a larger entity.

### 2.3. Finding Star Alleles

Star alleles denote a named haplotype of a gene, which may include one or more specific variants. They are normally numbered such that the second star allele for CYP2D6 is denoted CYP2D6*2. Suballeles also exist such as CYP2A6*4A. To extract these, we used the gene annotations from the aligned PubTator Central documents. We built a regular expression that searched immediately after gene mentions to identify instances of an asterisk followed by a word beginning with a digit (including colons to capture HLA alleles). We allowed for additional star alleles separated by white space, forward slashes, commas, and the words “and” and “or”. The normalized version of the variant is the name of the gene concatenated with the star-allele with whitespace removed. Furthermore we normalized the name of the allele specifically for the HLA family of genes. Specifically, we remove whitespace, leading zeros, and colons and then reinsert colons in character doubles. For example, HLA-B*05701 goes to HLA-B*57:01.

### 2.4. Filtering and Renormalizing Variants

PubTator Central uses the tmVar 2.0 tool for extracting variant mentions.^14^ It can extract DNA-level mutations (e.g. c.93G>A), protein-level mutations (e.g. T790M) and dbSNP IDs (e.g. rs12979860). Where possible, tmVar 2.0 will map the DNA/protein variants back to a dbSNP ID using co-mentions of genes in the document to filter down candidate matches. Hence a mention of PON1 alongside Q192R provides enough context to map it to rs662. We are primarily interested in those that can be mapped back to dbSNP IDs but kept all variant mentions as long as they contained a specific coordinate (i.e. filtering out cases such as c.G>A). For variants that are successfully mapped to a dbSNP ID by tmVar 2.0, PubTator Central does not provide the normalized text form of the variant. For example, ‘a PON1 glutamine-to-arginine mutation at residue 192’ is mapped successfully to rs662 but we also need the HGVS normalized form, p.Q192R. We implemented a re-normalization stage using regular expressions to capture the variant types output by tmVar 2.0.

### 2.5. Identifying Sentences of Interest

We extract all sentences that mention at least one variant (including star alleles) and one chemical from our filtered list. In an initial trial of this system focussing only on mentions of specific dbSNP IDs, we found that sentences were highly likely to mention pharmacogenomic events. However, when all variants were included, sentences were much less likely to discuss a pharmacogenomic association. Any machine learning system would then have a large challenge with such an imbalanced problem. We, therefore, filtered with a customized set of keywords (in supplementary materials) that increased the likelihood of a pharmacogenomic association or for a mention of a specific dbSNP ID.

### 2.6. Relation Classifier

The previous steps provide a large set of co-occurrences between variants and chemicals. But many of them are not describing a pharmacogenomic association. For instance, some of the sentences are very long and the chemical and variant are discussed in different contexts. Therefore we built relation classifiers to identify the pharmacogenomic relations.

Due to a class balance difference, we split the dataset into two. The first set is all potential chemical/variant where the variant is a specific rs ID or a star allele. The second set is all remaining chemical/variant associations which contain variants for specific DNA or protein changes. We worked on the two sets separately because, empirically, the first set has a higher likelihood for discussing pharmacogenomic associations. For each set, we annotated 500 sentences for pharmacogenomic associations. Chemical/variant associations were annotated as pharmacogenomic if the variant was discussed as physically interacting with the chemical, as affecting the metabolism, concentration or resistance of the chemical, as causing adverse events related to the chemical, as related to addiction to the chemical, or as part of a clinical test for that chemical use. The PharmGKB curators were also interested in variants found to be negatively associated pharmacogenomically with a chemical. Thus, we also annotated negative discoveries as positive sentences in our data set. For instance, “MDR1 C3435T does not affect the metabolism of telmisartan” would be annotated as pharmacogenomically associated. In total, we had 1000 annotated sentences which for evaluation are split into 80%/20% training and test sets. We include statistics on the annotated dataset in the supplementary materials.

We then created two Kindred relation classifiers,^24^ one for each for the two sets. We used a logistic regression as the classifier using Kindred’s default set of dependency-path based features. The logistic regression provides a thresholdable output score to control the precision-recall tradeoff. As in previous work, we used a high-precision/low-recall classifier.^11^ We created precision-recall curves to evaluate the trade-off and select a threshold of 0.75 for both classifiers. We filtered out cancer-associated chemicals (using DrugBank categories) that are associated with a protein/DNA variant as they are likely somatic events which are outside the focus of the PharmGKB resource.

### 2.7. Application to the entire literature

We then used the PubRunner infrastructure to apply these two classifiers across all the aligned sentences.^25^ This enabled the use of a compute cluster to quickly classify sentences as to whether they contain pharmacogenomic information. We then outputted relations along with the normalized form of the chemical and genes and other metadata. The gene information was included for star alleles and for variants normalized to dbSNP if it could be extracted from dbSNP. Metadata from the source paper is also included such as PubMed ID, title, journal, publication year and section within the paper. We also provide a version of the sentence which has HTML characters escaped and the entities in the relation highlighted with basic HTML tags for easy viewing. This output is the unfiltered version available.

We filtered relations that were scored lower than the previously selected threshold of 0.75 to reduce the false positive rate to create the pgxmine_filtered.tsv file. Finally, we collated the results by variant, chemical and where applicable gene IDs to find the total number of papers that discuss each variant/chemical association. This provides a rough metric of the importance of each association and allows a ranking to see which frequently discussed associations should be curated into PharmGKB. This collated version is released as the pgxmine_collated.tsv file.

### 2.8. Mapping to PharmGKB

We mapped the PGxMine set of associations to those found in PharmGKB. PharmGKB already contains some mappings directly to MeSH IDs but these needed to be supplemented with additional mappings from other sources. DrugBank provides a mapping from the MeSH ID used by PubTator Central to PharmGKB chemical IDs. Variants that have been mapped to dbSNPs or are star alleles can be mapped directly into PharmGKB. PharmGKB contains compound associations where multiple chemicals are associated with a variant. For simplicity, we unfold these associations so that all associations are from one chemical to one variant, allowing matching against the PGxMine data. For this comparison, we remove suballeles from star alleles (e.g. CYP3A5*3A -> CYP3A5*3). We also built a mapping for star alleles that map directly to a single rs ID to check if either was found in PharmGKB (e.g. POR*28 maps to rs1057868). Further details of this mapping are explained in the supplementary materials. For each PGxMine association, we then check if the variant has been seen in PharmGKB, the chemical has been seen in PharmGKB and whether this particular association has been seen.

### 2.9. Viewer

**Fig. 2.**
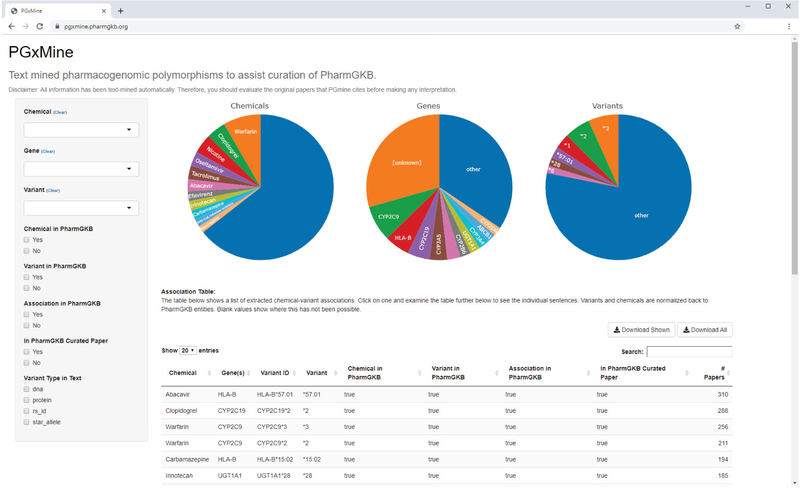
The data can be viewed through an R Shiny application in a web browser which allows the user to sort and filter the data and explore specific sentences that mention the association. The main table shows chemical/variant associations with gene information where possible. This table can be filtered using controls to the left to select specific chemicals, variants or genes. It can also be filtered for whether elements are already curated into PharmGKB. By selecting a row in the top table, a further table below (not shown) is populated with sentences that mention the selected association along with paper metadata.

The filtered and collated versions are then viewed through a viewer built using the R Shiny framework. This viewer (shown in Figure 2) shows the collated chemical-variant associations sorted in descending order by the number of papers that mention them. These results can then be filtered by a chemical, variant or gene, whether this data exists in PharmGKB and the type of variant, e.g. star allele. There are also Download buttons to facilitate downloading only a subset of the data. When a row in the top table is selected, a lower table is populated with all the sentences that discuss that particular association. These sentences, which may be from the same paper or across many papers, are ordered by publication date and additional metadata is provided for the source of the sentence with links to the original paper.

## 3. Results

### 3.1. Performance of Classifiers

**Fig. 3.**
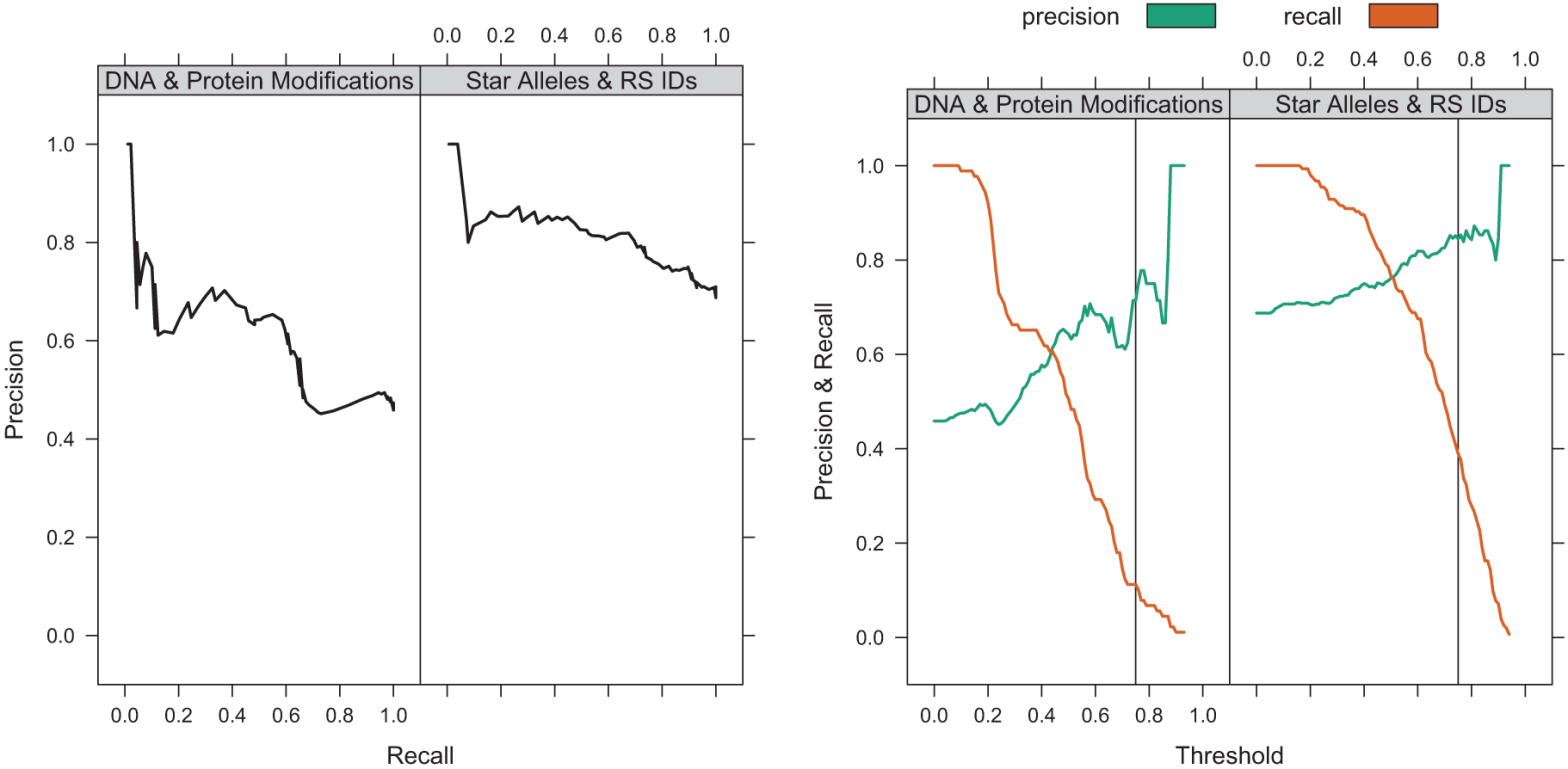
The precision-recall curves (above) and the same statistics shown against the threshold parameter for the classifier (below) for the two classifiers.

Using an 80%/20% training and test split, we evaluated the two classifiers independently. Figure 3 shows precision-recall curve plots of the three classifiers. The black line shows the selected high threshold (0.75) that provides higher precision (71.4% and 84.5% for the two classifiers) with the tradeoff of lower recall (11.2% and 39%).

### 3.2. Knowledge Base Results

By applying the two classifiers we identify 19,930 mentions of pharmacogenomic associations in 15,228 sentences across 7,170 papers. 41.1% of these associations were in the title or abstract. When collated, we find a total of 6,099 unique gene/variant associations. Of all the variant mentions, 39.9% can be normalized to an rs ID and 39.6% are star alleles.

As would be expected, there is a long-tail of associations with 75.6% of the unique associations being discussed in only a single paper. These singletons are of interest to the PharmGKB curators as the aim to improve coverage for rarer and less frequently discussed associations. We also find that the associations were found across all subsections of papers (Figure S2).

**Fig. 4.**
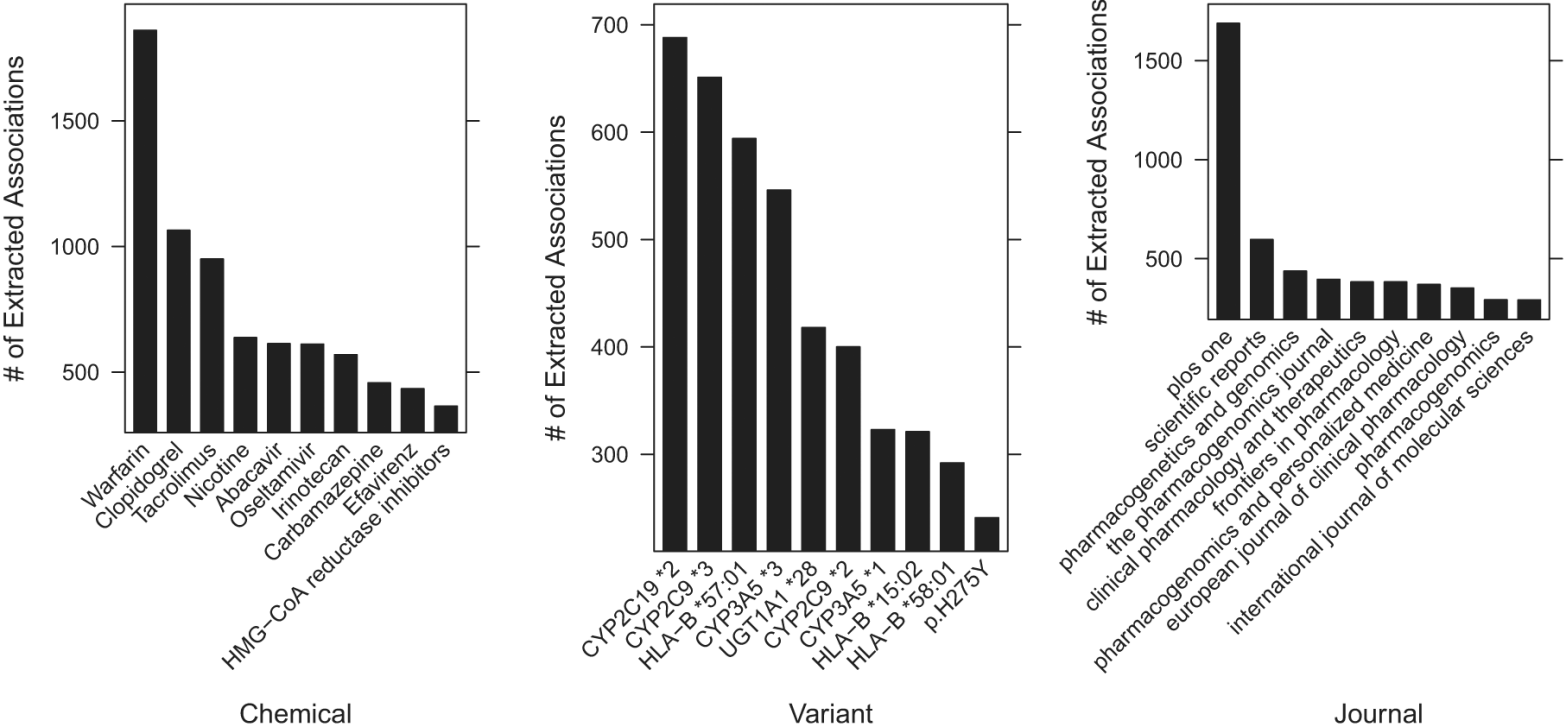
The 10 most common chemicals, variants and source journals by the number of extracted associations.

Figure 4 illustrates the most common chemicals in extracted chemical/variant associations as well as the most common variants with their gene names. Warfarin is the most frequently discussed drug in pharmacogenomic research and many of variants in the CYP family appear in the top ten variants. It also shows the most frequent journal sources that mentions are extracted from. Unsurprisingly, PLOS ONE is the most common source given the high number of papers that published there and also that they provide full-text articles.

Notably, a substantial number of the variants that cannot be normalized to a human gene appear to be HIV specific variants that appear in the virus genome and not the human genome. While these do affect drug resistance, they are outside the scope of PharmGKB but could be useful information for HIV researchers.

### 3.3. Comparison to PharmGKB

**Fig. 5.**
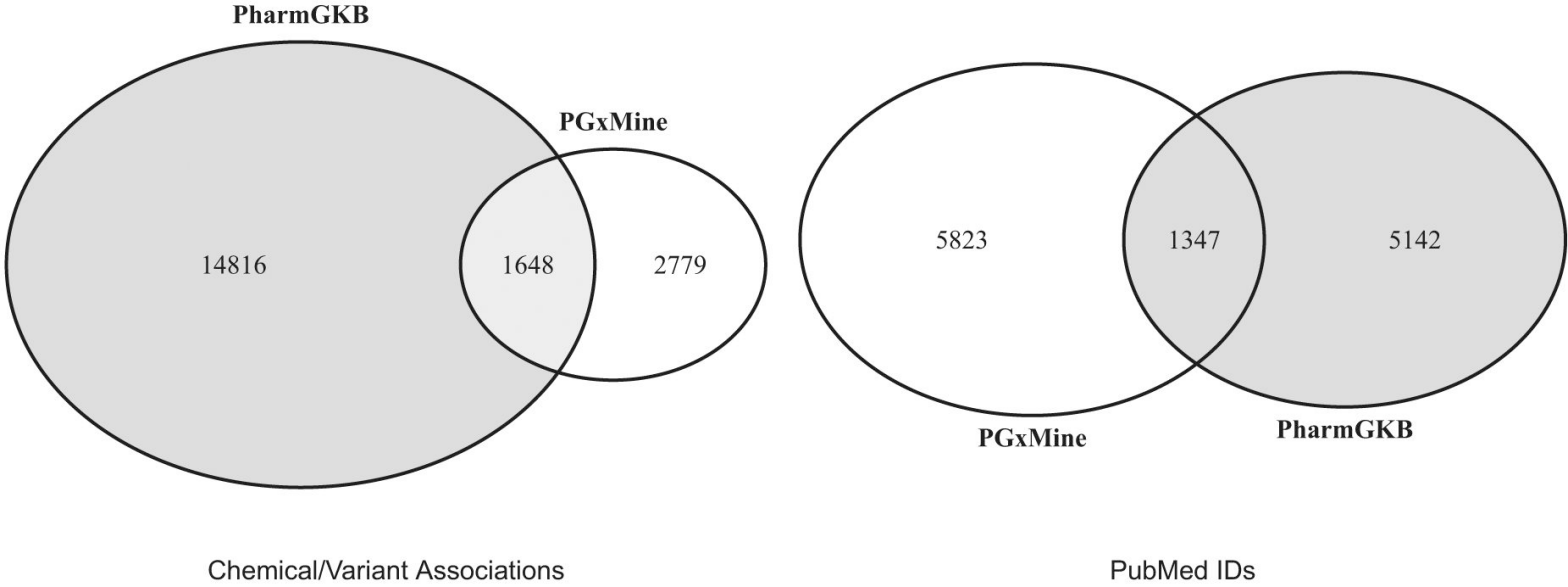
Venn Diagrams for the overlap in associations between PharmGKB and the PGxMine resource and papers identified in both resources.

We compared the associations extracted from the literature with the contents of the PharmGKB knowledgebase downloaded on 26 September 2019. Figure 5 shows the overlap of associations and specific papers. The scale of the PharmGKB knowledgebase cannot be understated and this figure illustrates the vast number of associations that have already been manually curated. 37.2% of associations found in PGxMine have previously been curated into PharmGKB but 2,779 could be curatable associations. As we identify mentions of associations, many of which will be in papers discussing previous work and not a new result, we wouldn’t expect to see a large overlap in the articles in PGxMine and PharmGKB. Nevertheless, we do see a sizeable overlap of papers based on PubMed IDs suggesting that PGxMine does often directly identify the appropriate paper for curation.

### 3.4. Utility for Curators

The goal of PGxMine is to lead the PharmGKB curators to new papers that should be curated. To evaluate this, the PharmGKB curators reviewed a subset of the top chemical/variant associations. The top 100 associations that were not in PharmGKB and did not appear in a previously curated paper were provided to the PharmGKB curators. They evaluated each association with the sentences and metadata provided by PGxMine. They judged whether the associated papers were appropriate for curation into PharmGKB or whether the discovered papers likely led to curatable papers. Of the 100 associations, 57 led directly to at least one curatable paper. A further 24 associations likely led to curatable papers through citations in the papers discovered by PGxMine.

Of the 57 that led directly to curatable papers, 37 led to a single curatable paper, 16 led to two curatable papers, 3 led to three papers and one association (Donepezil with CYP2D6 rs1080985) led to five papers for curation. In total, the 100 associations examined led directly to 83 curatable papers and likely 24+ other papers that could be found through examining citations of the PGxMine extracted papers.

The curators made use of the sentence metadata that identifies the section of the paper and found that associations mentioned in the Results section were more likely to be in curatable papers. Associations in the Introduction section were more likely to lead to valuable papers through citations and that specific paper would not be curatable. The curators normally focus on a smaller set of journals that publish pharmacogenomic studies. These results identified several papers in journals outside this group and show that PGxMine is a valuable tool to survey the broader biomedical literature without putting further burden on the PharmGKB curators.

## 4. Discussion

Our results suggest that PGxmine will assist PharmGKB curators but there are several notable limitations. The results of PGxMine rely entirely on the results of PubTator Central. We have explored running the individual NER tools on the large corpus ourselves but the computational cost, in terms of time and memory usage, is immense. Updates to our resource will, therefore, depend on the update frequency of PubTator Central which we hope to be frequent. This reliance on PGxMine on PubTator Central also means that we inherit any limitations of the NER tools used. While the NER tools are state-of-the-art for the entities that they are extracting, mistakes will still be made. We attempted to clean up issues by requiring longer terms and filtering out certain drug categories.

The main limiting factor is the poor access to full-text papers for text mining. 58.9% (11,735/19,930) mentions of pharmacogenomic associations were extracted from the main text of the article. Only a fraction of the biomedical literature is accessible for text mining. With only 12.3% (796/6,489) of the papers curated in PharmGKB having full-text available, it is understandable that PGxMine fails to catch many of the previously curated papers. PGxMine is also limited to associations found within the same sentence. This is a problem that all current information extraction systems face as the false positive rate explodes for systems that attempt to extract relations across sentences.^26^

The tradeoff of recall and precision is a challenging one for many machine learning problems. Curators become frustrated with a system that has too many false positives and are understanding of a system that does not have 100% coverage of associations. We, therefore, target high-precision low-recall for this problem, especially when the frequently discussed associations are found in hundreds of papers, meaning that they will likely be discovered by our pipeline. For example, the most frequently extracted association is Abacavir with HLA-B*57:01 in 272 papers. This means we should capture frequently discussed associations that aren’t yet curated into PharmGKB. We find that the vast majority of extracted associations (75.6%) are only mentioned in one paper. While more frequently discussed associations are of greater interest to PharmGKB, these singletons provide a valuable trove for further curation. The most common mistake that the classifier makes is incorrectly matching a drug with a variant in a different clause of a sentence. This occurs most frequently in sentences with multiple drugs and variants. Another challenge is capturing diplotypes of alleles or more complicated haplotype arrangements. While these are rare, they provide useful information for curators.

## 5. Conclusions

We have presented PGxMine, a knowledge base of pharmacogenomic associations to support curation of PharmGKB database. We developed a method for extracting knowledge from the new PubTator Central resource using a text alignment system coupled with a method for identifying star alleles and proven relation extraction methods. Our results can be updated regularly as PubTator Central is updated. We make the code and supplementary materials available with an MIT license (https://github.com/jakelever/pgxmine) and data available under CC0 license (https://doi.org/10.5281/zenodo.3360930) with the understanding that all data is extracted automatically and has not been vetted by the PharmGKB curation team.

## Supplementary Material

1
