# Supplementary material for: PGxMine: Text mining for curation of PharmGKB
Source: Pac Symp Biocomput. Author manuscript; Available in PMC 2020 Jan 1. (PMC6917032)
Supplement: 1 [file NIHMS1061502-supplement-1.pdf]

Supplementary Materials for PGxMine: Text mining for curation of PharmGKB

This file contains details of specific methods for the PGxMine project.

Text Alignment

PubTator Central provides a five-column file with mentions of different biomedical entities. An example is shown in Figure S1.

PubMed ID	Entity Type	Normalized ID	Text	Tool
29776386	Gene	79001	VKORC1	GNormPlus
29776386	Gene	8529	CYP4F2	GNormPlus
29776386	Gene	1728	NQO1	GNormPlus
29776386	Mutation	rs9923231	rs9923231	tmVar
29776386	Mutation	rs2108622	rs2108622	tmVar
29776386	Mutation	rs1800566	rs1800566	tmVar
29776386	Chemical	MESH:D014859	warfarin	TaggerOne

...

29776386:
Impact of **VKORC1** **rs9923231**, **CYP4F2** **rs2108622** and **NQO1** **rs1800566** SNPs on the daily stable **warfarin** dose

Figure S1. Data from PubTator Central is shown in the table and contains five columns for the PubMed ID, entity type, normalized ID, mention and tool used for extraction. The alignment of this example data is shown to text from the corresponding paper.

Drug lists

The list of drugs to extract and map is created by the createDrugList.py script. This uses MeSH, DrugBank and PharmGKB drug lists to create a mapping file from MeSH ID to PharmGKB ID. It also provides a normalized name using the DrugBank name, and flags whether the drug is a cancer treatment (if 'Antineoplastic' appears in a DrugBank category).

It removes drugs in categories: 'Elements','Adenine Nucleotides','Sweetening Agents','Salt Solutions','Supplements','Solvents','Electrolyte Solutions','Food Additives','Food','Lactates','Diluents','Gases','Mineral Supplements','Basic Lotions and Liniments','Phosphate salts','Potassium Salt'

It overrides filters to allow: 'fludarabine', 'nilotinib', 'diamorphine', 'cocaine', 'lopinavir', 'flucloxacillin', 'isoniazid', 'hydrochlorothiazide', 'tolbutamide', 'streptomycin', 'ampicillin', 'phenobarbital', 'azithromycin', 'tenofovir', 'peramivir', 'artemisinin', 'lapatinib', 'rociletinib'

It specifically removes the following chemicals: 'Cholesterol','Carbon dioxide','Adenine','Guanine','Thymine','Uracil','Cytosine','Adenosine','Guanosine','5-Methyluridine','Uridine','Cytidine','Deoxyadenosine','Deoxyguanosine','Thymidine','Deoxyuridine','Deoxycytidine','Heparin','Hydrocortisone','Estradiol','Tretinoin','Testosterone','Progesterone','Melatonin'

It also removes chemicals without any product names or FDA labels. To supplement DrugBank and PharmGKB MeSH mappings, it tries exact matching against MeSH terms with the name of the chemical, and the name of the chemical + 'hydrochloride'.

Identifying Sentences of Interest

The [findPGxSentences.py]() finds star alleles using a regular expression and then parses the document to find sentences that mention at least one chemical and at least one variant (including protein/DNA modifictions, rsIDs, and star alleles). It filters out sentences by requiring that one of the below substrings is found in the sentence or that an rsID is found. These strings are from pgx_filter_terms.txt.

- associat
- response
- study
- studies
- significan
- survival
- compare
- observe
- interaction
- efficacy
- toxicity
- metabol
- resistan
- sensitiv
- marker
- correlate
- outcome
- susceptibility
- pharmaco
- predict
- efflux

Mapping rsIDs to gene names using dbSNP

In order to show gene names, rsIDs were mapped to them using dbSNP. The [linkRSIDToGeneName.py]() script parses the VCF download of dbSNP. It uses the PubTator Central annotations to check with rsIDs need to be saved so that the entirety of dbSNP is not saved.

Mapping star alleles to rsIDs (where appropriate)

Some star alleles contain only a single mutation and are represented by an rsID (e.g. POR*28 to rs1057868). Most star alleles are more complex haplotypes of a series of mutations in a genes. No mapping exists for all star alleles, though some exist for some gene familes (e.g. PharmVar). The linkStarToRSID.py script does a regular expression search of the biomedical literature to find occurrences of star alleles right next to an rsID, e.g. "We study POR*28 (rs1057868)" with the rsID in brackets. We then manually filtered this list to produce starRSMappings.tsv.

Annotated data sets

We annotated two sets of 500 sentences for relations between drugs and variants. The first 500 sentence set was "Star Alleles & RS IDs" that contain sentences with star alleles (e.g. CYP2C19*2) or specific dbSNP RS IDs (e.g. rs9923231) and has a higher likelihood of containing pharmacogenomic information. The second 500 sentence set was "DNA & Protein Modifications" and contains sentences which mention specific DNA (e.g. c.521T>C) or protein modifications (e.g. p.C3435T). It had a lower class balance. The table below shows the number of relations in the two sets as associated or not associated.

Relation Type	Star Alleles & RS IDs	DNA & Protein Modifications
Pharmacogenomically associated	412	206
Not Pharmacogenomically associated	201	472

The datasets are available in the Github repo in the two archives: annotations.variant_star_rs.bioc.xml.gz and variant_other.bioc.xml.gz

Subsections

The figure below shows the sections of the full-text papers from which the mentions are extracted. All sections are covered with the discussion sections being the more common area for discovery. The section headers are extracted by PubRunner using a custom list of section headings and then grouped into the categories shown in the figure.

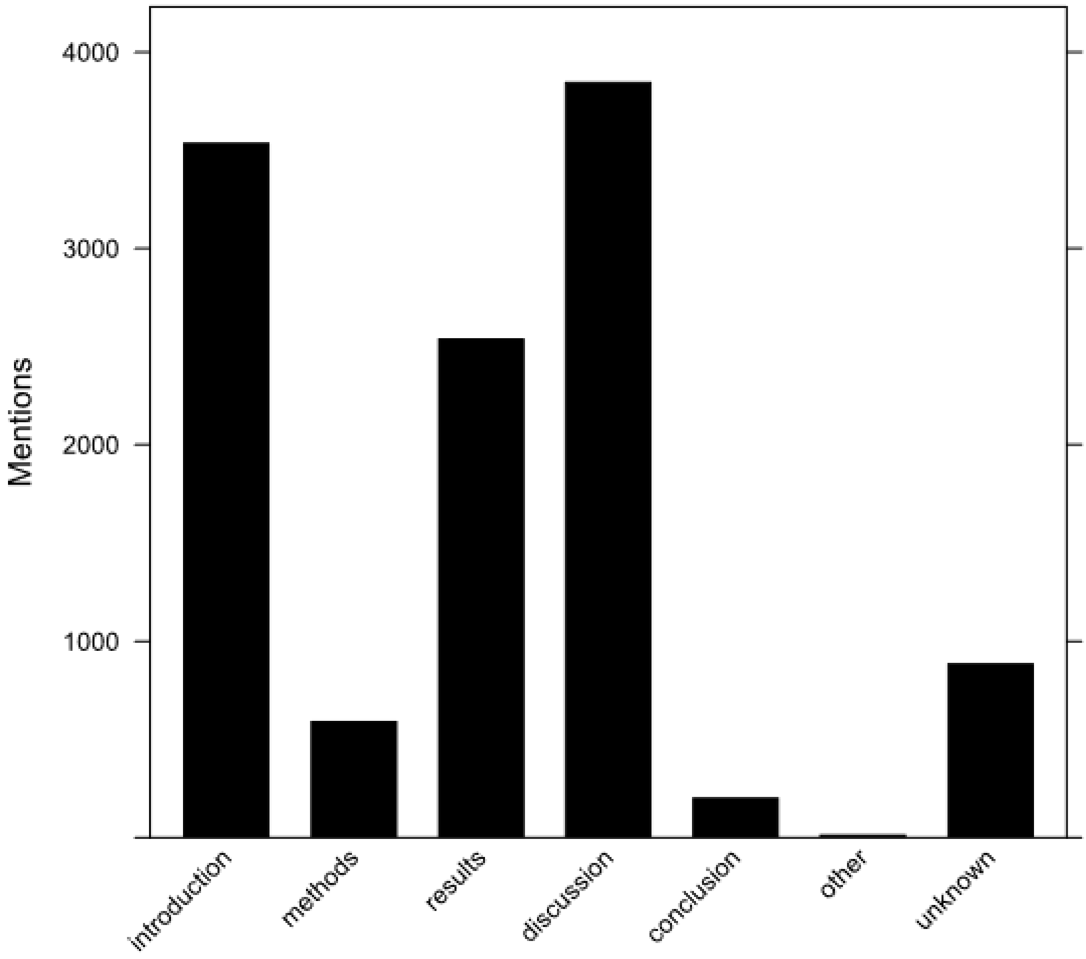

Figure S2. Number of mentions of pharmacogenomic associations extracted from each subsection of full-text papers.
